# Assessment of a Side-Row Continuous Canopy Shaking Harvester and Its Adaptability to the Portuguese Cobrançosa Variety in High-Density Olive Orchards

**DOI:** 10.3390/s23031740

**Published:** 2023-02-03

**Authors:** Fernando Aragon-Rodriguez, António B. Dias, Anacleto Pinheiro, José Peça, Ivo Lourenço Días, Sergio Castro-Garcia

**Affiliations:** 1 Department of Rural Engineering, E.T.S.I.A.M, University of Cordoba, Campus de Rabanales, Ctra. Nacional IV Km 396, 14014 Cordoba, Spain; 2 MED—Mediterranean Institute for Agriculture, Environment and Development & Departmento de Engenharia Rural, Escola de Ciências e Tecnologia, Universidade de Évora, Pólo da Mitra, Ap. 94, 7006-554 Evora, Portugal

**Keywords:** canopy shaker, harvesting, olive, Cobrançosa, vibration

## Abstract

The olive tree is an important crop in Portugal, where different levels of intensification coexist. The traditional olive orchards present profitability problems, mainly due to harvesting, so there has been a drastic reconversion towards high-density or super-high-density olive orchards. The latter present major constraints due to very specific needs for their use, being practically destined for new orchards. Consequently, the possibility of using systems based on canopy shakers in high-density olive orchards with local varieties is promising. The objective of this work is to evaluate a prototype canopy shaker for the harvesting of high-density olive orchards of the Portuguese variety ‘Cobrançosa’. The evaluation is based on the study of canopy shaking in order to adapt canopy training and the adaptability of the machine. For this purpose, the vibration of 72 points of the tree canopy was recorded and a qualitative assessment of the harvest was carried out. Differences were found between the different zones according to the direction of the forward movement of the harvester and the distance to the trunk. These differences were associated with the values obtained for fruit detachment, and a greater quantity of fruit was harvested in the areas of the canopy in contact with the rods.

## 1. Introduction

The olive tree (*Olea europaea* L.) is an age-old crop in the countries in the Mediterranean basin and plays a major role in the economy and the preservation of the territory and landscape of southern European countries such as Portugal [1,2]. Portugal currently ranks ninth in terms of olive production and cultivated olive orchard area, with 722,580 t and 379,440 ha, respectively [3]. Different cultivation systems coexist in the country, such as traditional olive orchards, high-density olive orchards (HD) and super-high-density olive orchards (SHD), which cover 37.2%, 33.2% and 29.6% of the area, respectively [4]. The traditional olive orchard is characterised by a large planting frame and low tree density. There is a wide variation, with between 30 and 180 trees ha^−1^, and the average density in Portugal is 100 trees ha^−1^ [5]. Furthermore, each tree is several feet tall and large in size, and since it is generally rainfed, it produces low yields [6]. Harvesting is difficult in traditional olive orchards owing to the complex structure and geometry of the trees, which leads to high harvesting costs [7]. Harvesting in the traditional multiple trunk orchards is performed mainly by means of manual methods using long poles or sticks, which may or may not be combined with the use of hand-held harvesting systems [8]. In the single trunk traditional orchards, farmers can carry out olive harvesting using mechanised solutions such as trunk shakers with an inverted umbrella [9]. The lower profitability of these orchards has contributed to their conversion into more intensive plantations, which are less dependent on manual labour and have higher production levels. This induces traditional orchards with suitable topography and water conditions, with flat or gently sloping land and the possibility of irrigation, to become more intensive orchards [10]. This conversion has been marked in the last decade in Portugal, with a 10% reduction in the area devoted to traditional olive orchards [4].

Intensive olive orchards can be harvested by means of trunk shaking (using trunk shakers) or canopy shaking (using canopy shakers or straddle harvesters) [11]. The most widespread method used for mechanised harvesting in intensive olive orchards is the trunk shaker [12], which is based on the application of a forced vibration to the trunk that is transmitted to the branches and detaches the fruit [13]. However, there are problems with the efficiency of trunk vibrators during mechanised harvesting if plantations have been intensified by increasing the density of trees, as they are discontinuous systems, and this is noticeable from 400 trees ha^−1^ upwards [6]. This method also has a number of other drawbacks, such as limited canopy size, the risk of debarking the trunk, low harvesting efficiency in certain varieties and high labour requirements.

Canopy shaker and straddle harvester systems have the advantage of working continuously and unifying different basic harvesting operations. They may have structures for the interception, transportation, cleaning and storage of the fruit, in addition to the shaking system, which improves the efficiency of the process and reduces the need for manpower. They are based on the application of a forced vibration of the tree canopy through the use of rods that cause the fruit to detach [14]. Canopy shakers are more flexible when used with more traditional varieties and in different types of olive orchards with appropriate pruning and training, but their harvesting efficiency is lower when compared to that of straddle harvesters [15]. Straddle harvesters are mostly intended for use in new plantations formed of narrow hedgerows [16], in which specific varieties with lower vigour, early maturity and high productivity are cultivated [6]. The characteristics of current olive orchards and the versatility of canopy shakers make their use interesting, as they can be used to harvest traditional and high-density olive trees producing local varieties [8,17]. The use of local varieties also mitigates the genetic erosion effects produced when cultivating specific varieties (usually ‘Arbequina’, ‘Arbosana’ and ‘Koreneiki’), which require systems employing straddle harvesters [2,18,19]. This replacement of typically local varieties such as ‘Galega’ or ‘Cobrançosa’ with foreign, and particularly Spanish, varieties has been a significant factor, to the detriment of Portugal’s varietal heritage. The development of harvesting systems based on the canopy shaker should be aimed at mutual adaptation between the machine and the tree [20] in an attempt to minimise the weak points of this system, one of the principal ones being the efficiency of fruit detachment. The aim of this work is to evaluate a prototype of a continuous harvester for the harvesting of high-density olive orchards containing the Portuguese ‘Cobrançosa’ variety. This is carried out on the basis of studying canopy shaking in order to adapt the formation of the canopy and the adaptability of the machine.

## 2. Materials and Methods

The trials were conducted in an irrigated, high-density 13-year-old olive orchard (285 trees ha^−1^) with a tree spacing of 7 × 5 m producing the ‘Cobrançosa’ cultivar, located on Torre das Figueiras farm, Monforte (lat: 39°04′10″ N, long: 7°28′14″ W), in Portugal. The first step was the characterisation of the trees and fruits tested. For the characterisation of the trees, their geometry was established by measuring diameters, tree height and lower branches’ height, and canopy volume was estimated using the ellipsoid method [21]. Tree fruit production was also measured. The fruit was measured for its detachment force using a dynamometer (Carpo, 1000 GRAMM G, France), weighed using a precision scale (Gram-Group; GRAM SPX, Barcelona, Spain) and its ripening index was evaluated by employing the Jaén method [22]. The olives were subsequently crushed, and their moisture and fat contents were determined by means of near infrared spectroscopy (NIR) technology (FOSS, Olivia, Hillerod, Denmark). The characteristics of the trees and fruit at the time of the trials are shown in Table 1 and Table 2.

**Figure 1:**
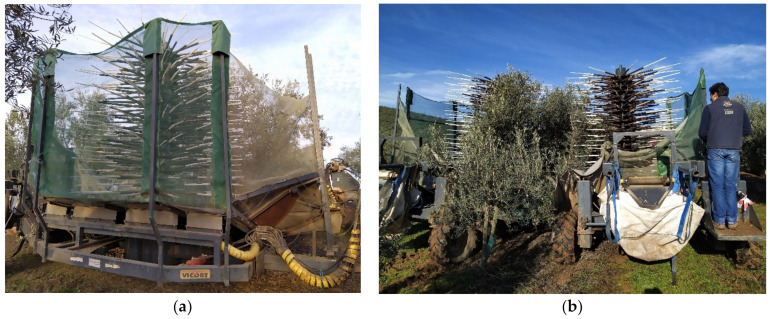
Prototype used for mechanised harvesting of high-density olive orchard. (**a**) Side view of the machine; (**b**) rear view of the machine.

The harvesting was carried out by employing a prototype developed by the company Vitor Cardoso Lda. and the University of Évora [23]. It is composed of two equal symmetrical trailed machines that are arranged on each side of the row of trees and move simultaneously, harvesting both sides (Figure 1). It comprises a shaking system, an interception platform and a recovery system that stores the fruit in a large bag. The olive branches are shaken by a vibrating rotor with 232 straight and flexible polyamide rods of variable sections with a total length of 1.2 m distributed at 21 levels, with an effective vibration depth ranging between 1.1 and 3.6 m. The vibration frequency can be modified by adjusting the power take-off (PTO) speed of the tractor. The prototype ground speed was 0.3 km h^−1^ and the working frequency was 9 Hz (at 540 cycles min^−1^).

**Figure 2:**
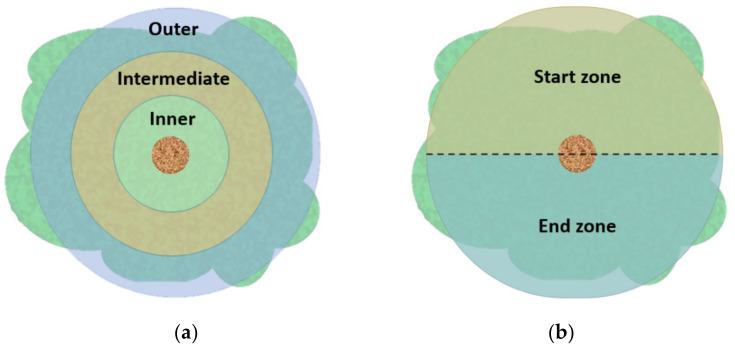
Division of the different study areas of the canopy. (**a**) Radial division of the zones; (**b**) division of the zones according to the direction of the forward movement of the harvester in the zones.

Twelve trees were randomly selected in the test plot and different canopy zones were established. The canopy was first divided radially into three zones: an inner zone (up to 0.7 m from the trunk), an intermediate zone (from 0.7 to 1.4 m from the trunk) and an outer zone (more than 1.4 m from the trunk) (Figure 2a). Then, another division was made according to the direction of the forward movement of the harvester: the start zone of the harvester and the end zone of the harvester (Figure 2b). To evaluate the performance of the prototype, a production class was defined based on the olive count: none (1), low (2), medium (3) and abundant (4), for each of the combinations of zones before and after harvesting with the prototype. After the mechanised harvest, the fruit of each tree was weighed, adding the fruit harvested by the machine and the fruit remaining on the tree.

Vibration was first measured at the end of the rods of the prototype in the workshop, i.e., working without a load and without harvesting the tree. It was subsequently measured at the canopy of the trees that had been previously selected for the qualitative analysis of their production. A triaxial MEMS accelerometer (Gulf Coast Data Concepts LLC X200-4, Waveland, MS, USA) with a measurement range of ±2000 m s^−2^, a 16-bit resolution, a sensitivity of 0.06 m s^−2^ and a sampling frequency of 400 Hz was used to record the vibration. There were 72 recorded points distributed on the main branches of the 12 trees, 6 for each of the trees, at different heights from 1.5 to 3.0 m from the ground and from the axis of the trunk up to 2 m on both sides of the tree. The position of the sensor in relation to the height of the tree and the longitudinal and transverse distance from the trunk axis were measured. The diameter of the branch on which the sensor was placed was also measured using a digital calliper (Mitutoyo; Absolute CD 20 DCX, Takatsu-ku, Kanagawa Prefecture, Japan).

The acceleration signals were analysed in the time domain using R v4.1.3 open software [24] and in the frequency domain using specific signal analysis software (OROS, NVGate v8.0, Koblenz, Germany), with a fast Fourier transformation (FFT) with 401 lines in a frequency range of 0–156.2 Hz with a 0.39 Hz resolution. Several variables were studied in the time and frequency domain.

The variables studied in the time domain were:

- Vibration time: time that elapsed between the first and the last event with a value over a resultant acceleration range. The beginning and end of the forced vibration on the tree branch was determined by measuring the vibration without any excitation except the action of natural phenomena in the 100 s before and after harvesting.
- Resultant acceleration (A_r_) (m s^−2^): vector sum of the three measurement axes (x, y, z) on each sensor.
- Peak acceleration (A_pk_): the 30 maximum values of peaks in resultant acceleration in the time domain, considering the sampling frequency and the vibration frequency.
- Crest factor: ratio between peak acceleration (A_pk_) and the RMS value of the resultant acceleration (A_r_).

The variables studied in the frequency domain were:

- Frequency: number of cycles of rod movement in the canopy per second (Hz).
- RMS acceleration (A_RMS_): vector sum of the root mean square (RMS) values of each accelerometer axis at the vibration frequency.

## 3. Results

### 3.1. Vibration Signals

The vibration recorded on the rods of the prototype working without a load attained mean A_RMS_ values of 127.5 m s^−2^ (SD = 15.6) and a median of 122.0 m s^−2^ (IQR = 19.6) when applying a drive frequency of 9.0 Hz (SD = 0.1). During harvesting, similar values of frequency, vibration time, RMS and peak acceleration were recorded at the tree canopy for the two symmetrical machines of which the prototype is composed, and there were no significant differences between them (Student’s *t*-test, *p* < 0.05; Mann–Whitney U test, *p* > 0.05) (Table 3). The mean working frequency value was 9.1 Hz (SD = 0.4). The working capacity of the machine was estimated by taking into account the forward speed of the prototype, with a value of 0.3 km h^−1^, and the planting frame of the plot, obtaining an estimated value of 0.13–0.15 ha h^−1^.

Significant differences were found as regards the vibration values between the area of the canopy at the entrance and exit of the machine, according to the forward direction of the harvester (Mann–Whitney U test, *p* < 0.05). Differences were also observed between the inner canopy zone and the rest of the zones, which showed no significant differences between them, for the entire height sampled (Kruskal–Wallis, *p* < 0.05; post hoc Mann–Whitney U test with Holm adjustment, *p* < 0.05). Table 4 shows the vibration values obtained for the canopy.

As can be seen in Table 4, there were differences among the vibration time, A_RMS_ and A_pk_, for the various zones according to the direction of the harvester or the distance to the trunk of the tree. These differences were more marked for A_RMS_ and A_pk_, with a moderate positive linear correlation between them (Pearson’s coefficient = 0.544, *p* < 0.05), than for vibration time, with all three variables following the same increasing or decreasing trend. These differences cancelled out between radial zones 2 and 3, and no significant differences were found for either.

Taking into account the A_RMS_ values measured in the rods and those obtained for the canopy (Table 1), the transmissibility of the acceleration between the prototype and the tree canopy ranged between 37.0% and 68.7%, depending on their radial location and their position with respect to the forward movement of the harvester. The lowest transmissibility values were located in the area closest to the trunk and the canopy end zone, and the highest in the area in which the harvester enters the canopy and the outermost part of the canopy. The A_pk_ values followed the same pattern, with values differing by 37.8–39.0% between these zones. The differences between zones for vibration time were more attenuated, with values of between 11.8% and 12.7%, although with significant differences. Most of the vibration times (80.6%) were maintained at a resultant acceleration of up to 100 m s^−2^, while 14.8% of vibration times were maintained at between 100 and 200 m s^−2^ and the remaining vibration times at values greater than 200 ms^−2^ of resultant acceleration.

There were no significant differences among the values attained for the shape measures of the vibration signal, crest factor, skewness and kurtosis, with respect to the resultant acceleration in the different canopy zones (Kruskal–Wallis, *p* < 0.05; post hoc Mann–Whitney U test with Holm adjustment, *p* < 0.05), with mean values of 7.5 (SD = 2.2), 4.1 (SD = 2.0) and 40.7 (SD = 42.3), respectively.

### 3.2. Evaluation of Mechanised Harvesting

**Figure 3:**
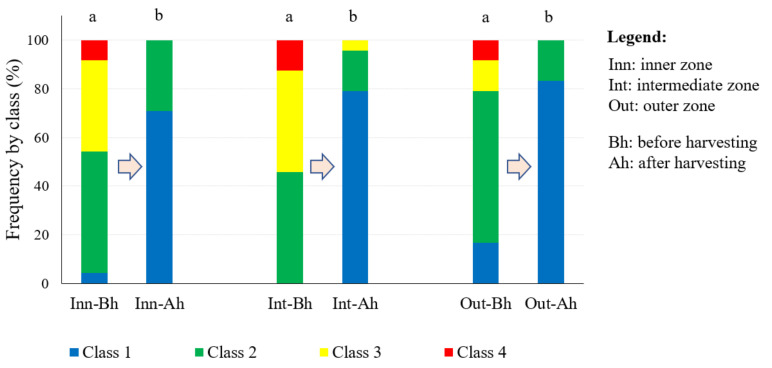
Qualitative comparison of quantity of fruit before and after mechanised harvesting in the different radial zones from the tree trunk in the study area (1.1 to 3.0 m above the ground). A different letter indicates significant differences (Friedman’s test, *p* < 0.05).

A qualitative analysis of the mechanised harvester was carried out by differentiating between the start and end zone of the prototype in the canopy and the different radial zones of the canopy (Figure 2). There were significant differences between the amount of fruit before and after mechanised harvesting in the three radial zones defined (Friedman’s test, *p* < 0.05), with a reduction in the amount of fruit after harvesting (Figure 3). It can be observed that there was a higher quantity of fruit in the inner and intermediate zone of the canopy before harvesting. However, the amount of fruit after harvesting was similarly reduced, with class 1 (none) representing between 71% and 83% and class 2 (low) representing between 17% and 29%, with the highest amount of fruit being in the inner zone.

**Figure 4:**
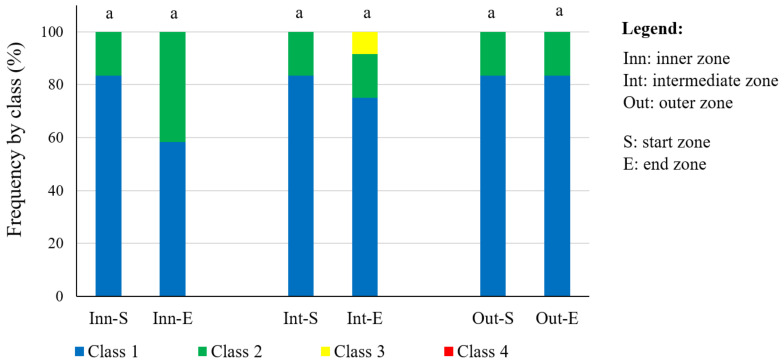
Qualitative comparison of fruit quantity after mechanised harvesting in the machine start and exit zone at the canopy of the tree in the study area (1.1 to 3.0 m above the ground) in different radial zones. A different letter indicates significant differences (Friedman’s test, *p* < 0.05).

The amount of fruit remaining on the tree in the canopy start and end zone according to the direction of the forward movement of the harvester was then assessed (Figure 4). The distribution of the amount of fruit was the same in the canopy start zone, regardless of the different radial zones, and 83% were class 1 (none) and the remainder were class 2 (low). No significant differences were found (Friedman’s test, *p* > 0.05) as regards the amount of fruit in the canopy end zone. However, there was a gradual decrease in the amount of fruit from the inner to the outer zone in the canopy end zone, with class 2 (low) dropping from 42% to 17%.

Minor damage to the canopy and trunk was observed. During harvesting, slight leaf fall and some minor bark detachment from the branches, caused by the machine’s rods, were observed, with no implications for the tree’s structure or future production.

## 4. Discussion

The A_RMS_ values obtained for the prototype rods working without a load were similar to those obtained by other authors in olive orchards when using canopy shakers [8]. However, they were below the values achieved when using straddle harvesters [25]. The frequency value generally used with canopy shakers is low, in the 3–5 Hz range, with high amplitude values of 100–200 mm [10,26,27], and the frequency value used is more typical of straddle harvester-based systems [25,28]. However, different vibration patterns can be employed by applying combinations of frequency and amplitude that result in high harvesting efficiency [29]. Some authors, in their work [30], designed and tested a canopy shaker with two independent sections that applied different frequency combinations to different areas of the tree with the aim of minimising tree damage and maximising harvesting efficiency. Another parameter of interest is the acceleration transmissibility ratio, which is defined as the value of the acceleration received by the tree canopy in relation to that supplied by the harvesting machine. In this work, an average value of 52.9% was obtained, similar to that obtained in olive orchards using a straddle harvester, with a value of 58.2% [25]. However, in the case of the straddle harvester there was less variation in this value across the canopy when compared to a greater oscillation recorded using the prototype described herein. This is owing to the mode of action of the harvesting system and the design of the plantation. In the case of the straddle harvester, the hedge resembles a flat two-dimensional system [31], in addition to which the machine compresses the tree along the tunnel, signifying that there is no difference between the horizontal areas of the canopy, although there is a difference between the vertical areas [25]. In this study, no differences were found between the study area as regards canopy height when using the canopy shaker, possibly because there was no such difference or perhaps because of the limited canopy height (between 1.1 and 3 m). Nevertheless, there were differences between the outer and inner canopy zones, as has been reported by other authors when using a canopy shaker with citrus fruit, and this is defined mainly by the penetration of the rods, which defines the zone of action of the shaking system [27]. This acceleration value was lower as we went deeper into the canopy owing to the vibration damping effect generated by the leaves [32]. This reduced its value by 36.4–40.8%, with values similar to the 42% and 43% obtained by various other authors [27,33]. In contrast, other authors [20] observed that the transmission of acceleration in the tree when using a trunk shaker in high-density olive orchards went from the grip zone in the trunk, along the trunk and, subsequently, from the inner to the outer branches, and that its value decreased from the inner zone to the outer zone of the canopy.

The vibration time is influenced principally by the forward speed of the machine and the characteristics and geometry of the canopy. This vibration time was 3.3 to 3.5 times higher than the values reported by other authors when using a canopy shaker, and was slightly higher in the outer areas of the canopy [27]. The distribution of the resultant acceleration values as regards the vibration time were very similar to those obtained in olive orchards using a canopy shaker and a straddle harvester, with a negative exponential distribution and a large number of low acceleration values, followed by a small percentage of high acceleration values [8,25]. This distribution of resultant acceleration in the vibration time was similar in the different areas of the canopy, as indicated by the lack of differences among the shape measurements. The vibration signals, therefore, followed a scaling factor depending on the canopy zone in which the vibration occurred. There was a lower presence of impacts for these signals, as indicated by the crest factor value, when compared to the shaker combs used for olive harvesting, which have a similar resultant acceleration distribution [8,34]. The low forward speed, which was outside the usual values of 0.8 to 1.5 km h^−1^ employed with lateral canopy shakers, not only increased the vibration time, but also significantly reduced the working capacity of the values of 0.25–0.50 ha h^−1^ indicated by other authors [26,27,35,36]. Although the design of the prototype confers a higher working capacity by providing a single pass, thus avoiding the need for the two passes required by lateral canopy shakers in order to harvest both sides of the tree, this was not sufficient to achieve the aforementioned working capacities. However, this design improves fruit interception by reducing the loss of fruit on the non-vibrating side of the tree, and the low forward speed facilitates the increased efficacy of fruit detachment [37,38].

The fruit was located mainly in the intermediate zone of the canopy, as has been reported for olive orchards by other authors [39]. There was little fruit in the canopy after harvesting, and a high number of olives were detached. The fruit remaining in the canopy was decreasingly distributed from the inner to the outer zone of the canopy, and the zones were associated with more fruit with a lower vibration time and A_RMS_ values (Table 4), as reported by other authors [27]. The differences between the quantity of the fruit in the canopy start and end zones after harvesting were not significant. This may, among other aspects, owe to the state of ripeness of the olive, which was high. However, in the canopy end area, a greater quantity of fruit was observed in the inner and intermediate zones when compared to the outer zone. These differences may increase with more immature stages of the olive, such as in the green state (Jaén index 0, 1 and 2), in which the olive has a higher detachment force and index, thus making it more difficult to harvest [40]. In order to improve the amount of fruit detached in those more unfavourable zones, it is important to facilitate the contact of the rods with the fruiting branches, which is influenced by the formation and architecture of the tree [41,42]. Hedge dimensions are a very important factor, as canopy shakers do not produce good vibration transmission, and their height and width must, therefore, be limited in order to achieve high fruit detachment values. The height is conditioned by the length of the shaker head and the width by the contact area of the rods according to their geometry [20,43]. Pruning should also limit fruit production in the inner zones and favour fruit production in the outer zones, where there are higher acceleration values. Another important aspect of the hedge is the achievement of continuity in the canopy of the trees and a uniform vertical contour by eliminating irregular branches that hinder the access of the rods, thus improving the efficiency of the process [42]. The angle of the branches should either facilitate contact with the rods or vary the design of the rods by providing them with a certain angle with which to facilitate entry and exit, along with the detachment of the fruit [44]. This training of the tree in order to adapt it to the harvester can initially be carried out by means of manual or mechanised pruning, and subsequently by employing mechanised maintenance pruning in such a way as to reduce the labour and costs of the operation, without affecting the productive level of the high-density olive orchard [45].

## 5. Conclusions

Advances in olive growing are based on the development of new, more technology-intensive planting systems, in which the different cultivation operations are mechanised, thus maintaining the profitability and competitiveness of the olive orchard. Harvesting is the most important of these operations and is, therefore, a priority area for research and development. It is also important to consider the structural design of the pre-established orchards and the use of traditional local varieties to carry out this mechanisation. This study has provided several results on the behaviour of a high-density olive orchard containing trees of the Portuguese ‘Cobrançosa’ variety when subjected to forced vibration using a canopy shaker, along with the adaptability of both elements through the pruning and training of the trees and the regulation of the machinery.

## Figures and Tables

**Table 1:** Characterisation of the trees on the trial plot (*n* = 12).

Tree Height (m)	Height from Ground to Canopy (m)	Canopy Height (m)	Perpendicular Diameter Tree Line (m)	Diameter at Tree Line (m)	Canopy Volume (m^3^) ^3^	Production per Tree (kg)
3.7 ^1^ (0.2) ^2^	0.9 (0.1)	2.9 (0.2)	3.6 (0.4)	3.9 (0.5)	21.0 (4.8)	22.0 (4.5)

^1^ Mean; ^2^ standard deviation; ^3^ applying the ellipsoid method [21].

**Table 2:** Characterisation of the fruit on the trial plot (*n* = 100).

Fresh Weight (FW) (g)	Detachment Force (DF) (N)	Detachment Index (DF/FW)	Ripening Index ^3^	Oil Content (% Wet Weight)	Oil Content (% Dry Weight)	Moisture Content (%)
3.7 ^1^ (0.7) ^2^	2.9 (0.3)	0.8 (0.2)	4.2 (0.1)	17.1 (1.5)	38.6 (1.8)	55.8 (2.0)

^1^ Mean; ^2^ standard deviation; ^3^ according to the Jaén method [22].

**Table 3:** Vibration parameter values for the two symmetrical machines of which the prototype is composed.

	Frequency (Hz)	Vibration Time (s)	A_RMS_ (m s^−2^)	A_pk_ (m s^−2^)
Right symmetrical machine	9.22 (0.25) ^1^ a ^3^	50.38 (9.91) ^2^ a	66.97 (40.67) ^2^ a	672.6 (468.1) ^2^ a
Left symmetrical machine	9.10 (0.34) ^1^ a	48.04 (11.66) ^2^ a	59.24 (44.75) ^2^ a	585.8 (552.1) ^2^ a

^1^ Mean values (standard deviation). ^2^ Median values (interquartile range). ^3^ Differences between letters in the same column indicate significant differences (Student’s *t*-test, *p* < 0.05; Mann–Whitney U test, *p* < 0.05).

**Table 4:** Vibration parameter values according to the direction of the forward movement of the harvester and the distance to the trunk.

		Vibration Time (s)	A_RMS_ (m s^−2^)	A_pk_ (m s^−2^)
According to the direction of the forward movement of the harvester	Start zone	53.11 (9.96) ^1^ a ^2^	83.80 (38.03) a	846.0 (522.8) a
	End zone	46.85 (9.81) b	53.39 (42.54) b	526.1 (445.0) b
According to the distance to the trunk	Inner zone	45.18 (9.65) a ^3^	45.11 (40.45) a	442.1 (456.5) a
	Intermediate zone	50.88 (7.42) b	70.97 (42.98) b	712.6 (371.1) b
	Outer zone	51.77 (12.14) b	76.25 (25.53) b	724.5 (159.5) b

^1^ Median values (interquartile range). ^2^ Differences between letters in the same column and section indicate significant differences (Mann–Whitney U test, *p* < 0.05). ^3^ Differences between letters in the same column and section indicate significant differences (Kruskal–Wallis, *p* < 0.05; post hoc Mann–Whitney U test with Holm adjustment, *p* < 0.05).

## Data Availability

Not applicable.
